# Metabolic profiling of induced acute pancreatitis and pancreatic cancer progression in a mutant Kras mouse model

**DOI:** 10.3389/fmolb.2022.937865

**Published:** 2022-08-25

**Authors:** Tatiana J. Carneiro, Joana Pinto, Eva M. Serrao, António S. Barros, Kevin M. Brindle, Ana M. Gil

**Affiliations:** ^1^ CICECO - Aveiro Institute of Materials (CICECO/UA), Department of Chemistry, University of Aveiro, Aveiro, Portugal; ^2^ Cancer Research UK, Cambridge Institute, University of Cambridge, Cambridge, United Kingdom; ^3^ Department of Biochemistry, University of Cambridge, Cambridge, United Kingdom

**Keywords:** pancreatitis, pancreatic cancer, PanIN, pancreatic ductal adenocarcinoma, KRAS, metabolomics, nuclear magnetic resonance (1H-NMR)

## Abstract

Untargeted Nuclear Magnetic Resonance (NMR) metabolomics of polar extracts from the pancreata of a caerulin-induced mouse model of pancreatitis (Pt) and of a transgenic mouse model of pancreatic cancer (PCa) were used to find metabolic markers of Pt and to characterize the metabolic changes accompanying PCa progression. Using multivariate analysis a 10-metabolite metabolic signature specific to Pt tissue was found to distinguish the benign condition from both normal tissue and precancerous tissue (low grade pancreatic intraepithelial neoplasia, PanIN, lesions). The mice pancreata showed significant changes in the progression from normal tissue, through low-grade and high-grade PanIN lesions to pancreatic ductal adenocarcinoma (PDA). These included increased lactate production, amino acid changes consistent with enhanced anaplerosis, decreased concentrations of intermediates in membrane biosynthesis (phosphocholine and phosphoethanolamine) and decreased glycosylated uridine phosphates, reflecting activation of the hexosamine biosynthesis pathway and protein glycosylation.

## Introduction

Pancreatic cancer (PCa) is the fourth leading cause of cancer-related deaths, with a 5-year survival rate of 5–6% (Siegel et al., 2011; Siegel et al., 2013), with over 80% of patients found to be ineligible for curative surgical treatment at the time of diagnosis (Ahlgren, 1996). Metabolomics or metabolic profiling of biological samples has emerged in recent years as a strategy for detecting aberrant metabolic behavior associated with cancer, and other diseases, with the possibility of revealing potential metabolic biomarkers to aid diagnosis and therapy follow-up (Madsen et al., 2010; Mamas et al., 2011; Emwas et al., 2013; Duarte et al., 2014; Wishart, 2016). The most common use of metabolomics is in the analysis of biofluids, either by Nuclear Magnetic Resonance (NMR) spectroscopy or mass spectrometry (MS)-based methods and, in the case of PCa, the subject has been reviewed recently (Turanli et al., 2021). Most metabolomic studies of PCa have addressed human serum/plasma and urine samples, with fewer reports on human saliva and pancreatic tissue extracts (Kaur et al., 2012; Di Gangi et al., 2014; Nguyen et al., 2015; Doug and Qingyue, 2017; Lindahl et al., 2017; Mayerle et al., 2018). Studies in animal models have considered either biofluids or pancreatic tissue, having focused on issues such as differentiating pancreatic ductal adenocarcinoma (PDA) from pancreatitis (Pt) or non-cancerous tissues (Fang et al., 2007; Yabushita et al., 2013; Nguyen et al., 2015), evaluating the effects of radiotherapy on tumor metabolite profile (He et al., 2013), comparing the metabolic characteristics of different xenograft models (Zhan et al., 2017), and seeking metabolic markers of PCa-induced wasting (Wyart et al., 2018) or of early pancreatic intraepithelial neoplasia (PanIN) and PCa progression (Fendrich et al., 2011; Wen et al., 2016; Schmahl et al., 2018). Genetically engineered mouse models of PCa, which recapitulate many aspects of the human disease, have been investigated for the metabolic changes that occur in pancreatic tissue during the progression of precursor lesions to PCa (Fendrich et al., 2011; Serrao et al., 2016; Schmahl et al., 2018; Vernucci et al., 2019). Positron emission tomography (PET) studies showed elevated glucose metabolism in precursor lesions in a mutant Kras model (Fendrich et al., 2011) and dynamic nuclear polarization (DNP) (Ardenkjaer-Larsen et al., 2003) enabled ^13^C magnetic resonance spectroscopic imaging measurements of hyperpolarized [1–^13^C]pyruvate metabolism showed increased lactate-labeling with disease progression (Serrao et al., 2016; Dutta et al., 2019). NMR profiling of pancreatic tissue in this mutant Kras model (Schmahl et al., 2018) showed increased levels of tyrosine, taurine, glucose and a number of unassigned resonances in the pancreas of 15-month old mice, where PanIN tissue was found to predominate. A recent comprehensive LC-MS/MS report evaluated intermediates in glycolysis, the pentose-phosphate-pathway (PPP), tricarboxylic acid (TCA) cycle, purine and pyrimidine metabolism, urea cycle and amino acid metabolism in an equivalent Kras mouse model in order to characterize metabolic changes taking place during PCa progression (Vernucci et al., 2019). This showed significant upregulation of glycolysis and PPP metabolites from the early stages of disease. Here we investigated whether untargeted NMR metabolomics could differentiate acute Pt from normal healthy tissue and early precancerous tissue, as well as determining the metabolic changes that accompany disease progression in the LSL-KrasG12D/+-p48Cre/+ (KC) mouse model of PCa. The metabolic signature found to describe pancreatic tissue affected by Pt, when corrected for the cellularity differences between Pt and normal and low-grade (LG) lesions groups, has the potential to aid in the diagnosis of acute Pt. The different stages of PCa progression were characterized from predominantly LG lesions (mPanIN 1 and 1A), through high grade (HG) lesions (mPanIN 2 and 3) to PDA, in order to build on previously proposed metabolic markers of disease progression.

## Materials and methods

### Animal model

Animal experiments complied with licenses issued under the Animals (Scientific Procedures) Act of 1986. Protocols were approved by the Cancer Research UK, Cambridge Institute Animal Welfare and Ethical Review Body. Table 1 lists the characteristics of the wild type (wt, C57BL/6) and genetically engineered mouse groups studied. Detailed descriptions may be found elsewhere (Olive et al., 2009; Serrao et al., 2016). Briefly, the control group (N, normal tissue, *n* = 7) comprised animals that did not develop lesions or tumors (C57BL/6 wt mice); the pancreatitis group (Pt, *n* = 6) comprised C57BL/6 wt mice in which acute pancreatitis was induced by six hourly intraperitoneal 50 μg/kg caerulein (Sigma-Aldrich, Dorset, United Kingdom) injections (Sakai et al., 2012; Serrao et al., 2016); the low grade lesion group (LG, *n* = 5) comprised 4-month old KC (LSL-Kras^G12D/+^ - p48^Cre/+^) mice with predominant LG PanIN lesions (mPanIN 1 and 1A); the high grade lesion group (HG, *n* = 7) comprised 9-month old KC mice with predominant HG PanIN lesions (mPanIN 2 and 3); the spontaneous pancreatic ductal adenocarcinoma (PDA, *n* = 6) and sarcomatoid (Sarc., *n* = 9) groups corresponded to 3–6 months old KPC (LSL-Kras^G12/D+^;LSL-Tpr53^R172H/+^;Pdx-1-Cre) mice (Serrao et al., 2016). Tumors were studied when they were less than 5 mm in diameter.

**TABLE 1:** Characterization of mice groups studied, including corresponding sample numbers (n), strain, age and average number of pancreatic cells.

Condition designation		*n*	Mice strain	Mice age/months	Average cell numbers (relative error)^a^
Control	*N*	7	C57BL/6 (WT)	1.5–2	1761 (19%)
Pancreatitis	Pt	6	C57BL/6 (WT)^b^	1.5–2	4454 (8%)
Low-grade PanIN lesions	LG	5	LSL-Kras^G12D/+^ - p48^Cre/+^	2–4	2794 (19%)
High-grade PanIN lesions	HG	7		9	2422 (7%)
Pancreatic ductal adenocarcinoma	PDA	6	LSL-Kras^G12/D+^	3–6	2318 (29%)
			LSL-Tpr53^R172H/+^		
Sarcomatoid carcinoma	Sarc	9			2958 (19%)
			Pdx-1-Cre		

a Average cell numbers counted microscopically on sets of representative samples of each group.

b Mice injected with six hourly intraperitoneal injections with 50 μg/kg (mouse weight) of caerulein (Sigma-Aldrich, Dorset, United Kingdom) to induce pancreatitis.

### Metabolite extraction and NMR spectroscopy

Mice were sacrificed by cervical dislocation and pancreatic tissue rapidly (within a maximum of 35 s) excised and freeze-clamped using liquid nitrogen-cooled tongs. Tissues (weights in the 60–120 mg range) were homogenized (*ca.* 200 mg/ml) in 50 mM HEPES (4-(2-hydroxyethyl)-1-piperazineethanesulfonic acid), 1 mM EDTA (ethylenediaminetetraacetic acid), 0.7% sodium deoxy-cholate, 1% Nonidet P-40, 0.5 M lithium chloride, pH 7.6, using a Precellys 24 homogeniser (Stretton Scientific, Stretton, United Kingdom) and then extracted in methanol:chloroform:water (Wu et al., 2008). High-resolution ^1^H NMR spectra were obtained at 14.1 T (25°C, pH 7.2) on a Bruker 600 MHz NMR spectrometer (Bruker, Ettlingen, Germany). The zgpr pulse sequence (Bruker library) was used with the following acquisition conditions: 90° pulse; 7.3 kHz spectral width; 4.5 s acquisition time; 32 k data points; 64 transients; and 12.5 s recycling time. Free induction decays were zero-filled to 64 k and multiplied by a 0.3 Hz exponential function prior to Fourier transformation. Proton chemical shifts were referenced to 5 mM 3-(trimethylsilyl)-2,2′,3,3′-tetradeuteropropionic acid (TSP; 0.0 ppm) added to the samples. Although total inter-scan times (17 s) enabled quantitative spectra to be obtained, the TSP intensity varied considerably between some of the spectra (perhaps due to intermolecular interactions), thus hindering absolute quantitation. Therefore, only relative metabolite contents were evaluated. Peak assignments were made by reference to the literature, to spectral databases (Bruker BBIOREFCODE2 database and the human metabolome database - HMDB (Wishart et al., 2018) and by using 2D NMR spectra (Total Correlation Spectroscopy-TOCSY, Heteronuclear Single Quantum Coherence- HSQC and J-resolved) recorded for selected samples.

### Statistical analysis

Multivariate analysis was applied to the spectra, excluding the water region (δ 4.7–5.0). 1D spectra were aligned using recursive segment-wise peak alignment (RSPA) and normalized either using spectral total area (to measure Tissue metabolic signatures), or using average cell counts obtained by visual analysis of the microscopic records of paraffin-fixed samples representative of each animal group (to measure Cellular metabolic signatures) (Table 1). Principal component analysis (PCA) and partial least squares discriminant analysis (PLS-DA) were performed upon unit variance (UV) scaling of the spectra (SIMCA-P 11.5; Umetrics, Umeå, Sweden). PCA and PLS-DA loadings were back-transformed by multiplying each variable by its standard deviation and colored according to each variable’s importance to projection (VIP) (Matlab 8.3.0, The MathWorks Inc.). For PLS-DA models, Monte Carlo cross-validation (MCCV) (7 blocks, 500 runs) was carried out with recovery of Q^2^ values (predictive power) and confusion matrices, providing classification rates (CR), specificity and sensitivity. For each model of interest, the relevant peaks identified in loadings profiles were integrated (Amix 3.9.5, Bruker BioSpin, Rheinstetten, Germany), normalized, and variations assessed by univariate analysis (Shapiro-Wilk test to assess data was normality, Student’s t or Wilcoxon tests for normally or non-normally distributed data, respectively; R-statistical software). Effect sizes and statistical significance (*p* < 0.05) were calculated for the relevant metabolites and false discovery rate (FDR) correction, based on the Benjamini and Hochberg method to adjust *p*-values for multiple comparisons (Benjamini and Hochberg, 1995; Berben et al., 2012). In some cases, statistical total correlation spectroscopy (STOCSY) was performed (Matlab 8.3.0) to aid peak assignment previously attempted by 1D/2D NMR, as described in the previous section (Cloarec et al., 2005).

## Results

### Metabolic profiling of pancreatitis compared to healthy pancreas and pancreas bearing low-grade (LG) PanIN lesions

**FIGURE 1:**
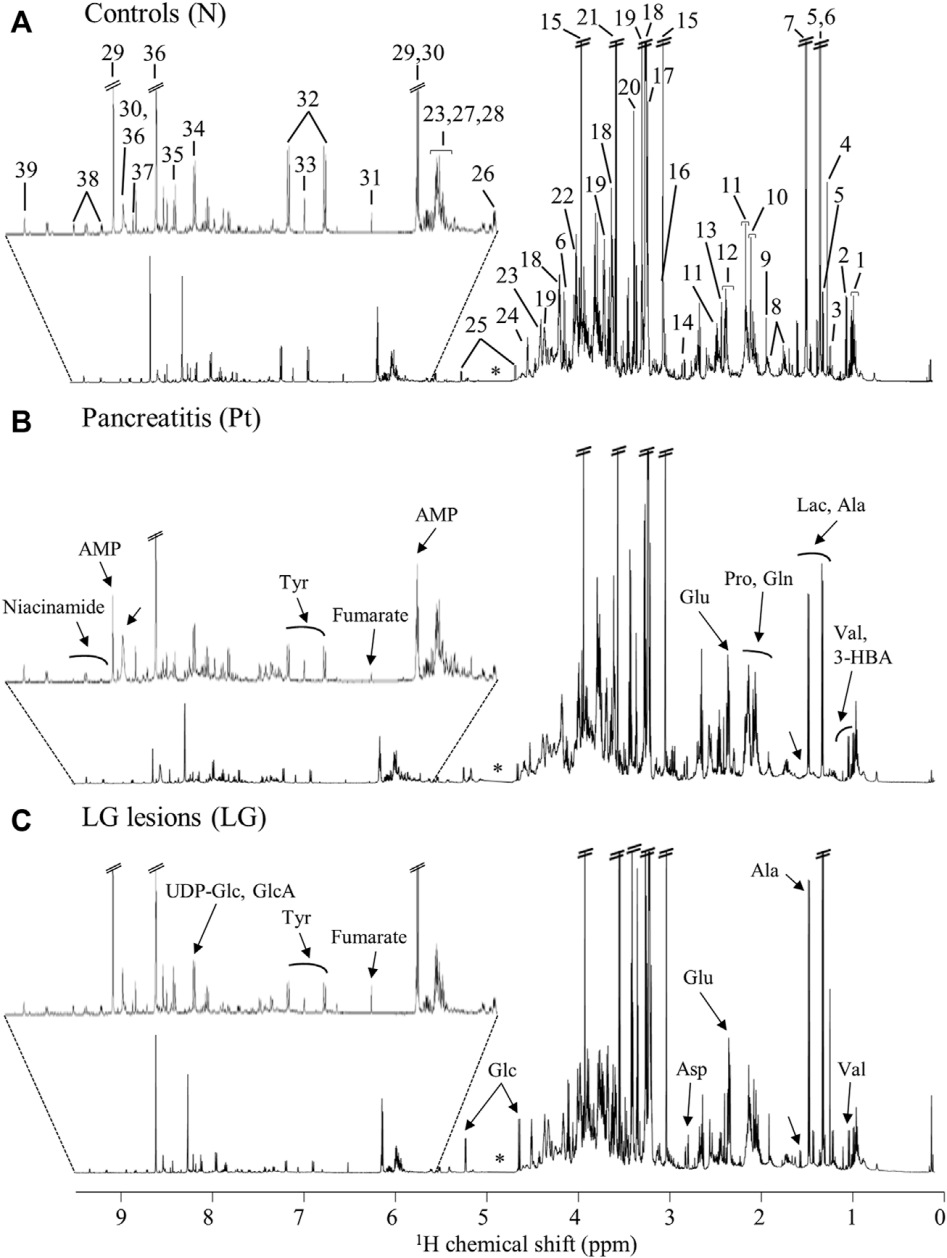
Average 600 MHz ^1^H NMR spectra of pancreatic polar extracts of **(A)** normal tissue (N), **(B)** tissue affected by induced acute pancreatitis (Pt) and **(C)** tissue bearing low-grade PanIN 1/1A lesions (LG). Main assignments: 1, isoleucine; 2, valine; 3, 3-hydroxybutyrate (3-HBA); 4, 3-hydroxyisovalerate (3-HIVA); 5, threonine; 6, lactate; 7, alanine; 8, lysine; 9, acetate; 10, proline; 11, glutamine; 12, glutamate; 13, succinate; 14, aspartate; 15, creatine; 16, creatinine; 17, choline; 18, phosphocholine (PC); 19, glycerophosphocholine (GPC); 20, methanol; 21, glycine; 22, phosphoethanolamine (PE); 23, uridine; 24, ascorbate; 25, glucose; 26, uridine diphosphate N-acetylglucosamine (UDP-GlcNAc); 27, uridine diphosphate (UDP); 28, uridine triphosphate (UTP); 29, adenosine monophosphate (AMP); 30, adenosine triphosphate (ATP); 31, fumarate: 32, tyrosine; 33, histidine; 34, UDP-glucose or glucuronate (UDP-Glc/GlcA); 35, uridine monophosphate (UMP); 36, adenosine diphosphate (ADP); 37, formate; 38, niacinamide (NAM); 39, nicotinamide adenine dinucleotide (NAD^+^). Arrows show visual spectral changes in Pt (middle spectrum) and in LG lesions (bottom spectrum), compared to normal tissue (top spectrum). ∗: cut-off water region.

**FIGURE 2:**
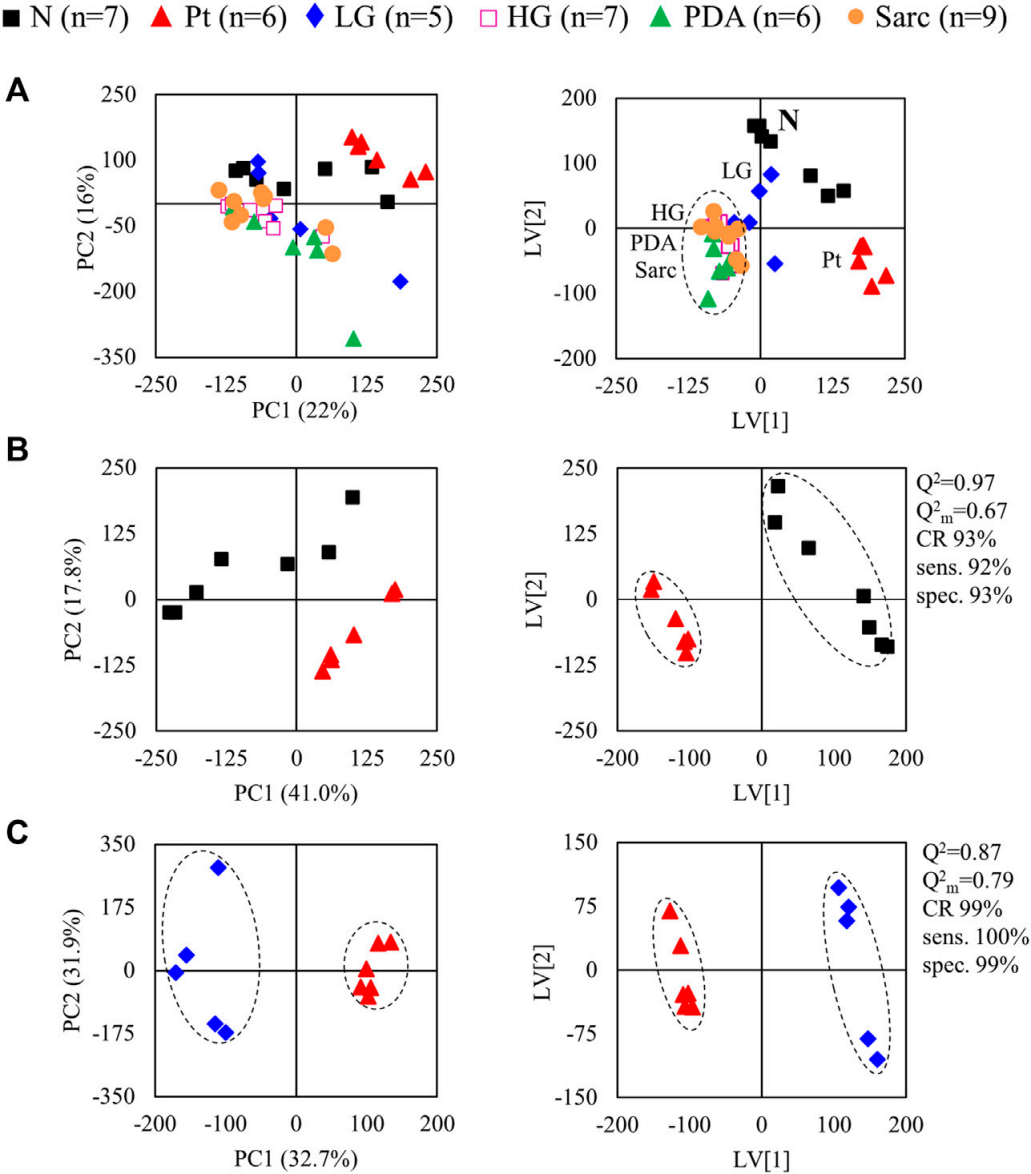
PCA (left) and PLS-DA (right) scores scatter plots for 1H NMR spectra (normalized to total area) of **(A)** all samples: controls, ■ N (*n* = 7), ▲ (red) pancreatitis, Pt (*n* = 6), ◆ (blue) LG PanIN lesions (*n* = 5), □ (pink) HG PanIN lesions (*n* = 7), ▲ (green) pancreatic ductal adenocarcinoma, PDA (*n* = 6), ● (orange) sarcomatoid carcinoma, Sarc (*n* = 9), and **(B)** Pt vs. Normal tissue, and **(C)** Pt vs. LG lesions tissue. Q^2^, predictive power of PLS-DA model; CR, classification rate; sens., sensitivity; spec., specificity.

The average ^1^H NMR spectra (sum of all spectra divided by the number of spectra) of pancreatic extracts from control C57BL/6 (WT) mice (N), wild-type animals with induced Pt (Pt) and KC mice with PanIN 1 and 1A lesions (LG) are shown in Figure 1. The complete list of identified metabolites in all samples may be found in Supplementary Table S1. The arrows in Figure 1 indicate some differences between Pt tissue or LG lesions (Figures 1B,C) compared to normal tissue (Figure 1A). As mouse ages were similar between N, Pt and LG groups (Table 1), age effects on metabolism are not expected to be a confounder in the comparison of these groups. Unsupervised multivariate analysis using PCA of the ^1^H NMR spectra of pancreatic extracts (after normalization to total spectral area) obtained for all animal groups (N, Pt, LG, HG, PDA and sarcomatoid) (Figure 2A, left) could distinguish mice with induced Pt from the remaining groups, while supervised PLS-DA analysis (Figure 2A, right) improved the separation between Pt, N and LG. The LG group partially overlapped with HG, PDA and Sarc groups, which clustered together. Despite the small number of samples, pairwise PCA and PLS-DA (and subsequent model validation by MCCV, Supplementary Table S2) confirmed the statistically robust distinction of Pt tissue from normal tissue (Figure 2B; Q^2^ = 0.97, Q^2^median = 0.67, 92% sens., 93% spec.) and from LG tissue (Figure 2C; Q^2^ = 0.87, Q^2^median = 0.79, 100% sens., 99% spec.). PLS-DA LV1 loadings plots of Pt vs*.* normal tissue (N) identified several metabolites that explain the group separation (orange/red signals in Supplementary Figure S1A). Analysis of these data revealed 22 identified metabolites and eight unassigned compounds that exhibited significantly different concentrations between Pt and N tissues (*p*-value < 0.05) (Table 2, *Tissue signature*). These differences remained significant after FDR correction (except for glycerophosphocholine (GPC), ^c^ in Table 2). The most significant differences in Pt samples compared to N samples (higher effect size and lower *p*-value, Table 2 and Volcano plot in Supplementary Figure S1B) comprised higher levels of ATP, ADP, glutamate, reduced glutathione (GSH), phenylalanine and taurine (and unassigned resonances U4 δ 5.16 and U6 δ 5.70); and lower levels of alanine, AMP, creatine, formate, histidine, niacinamide (NAM), tyrosine and uridine triphosphate-*N-*acetylglucosamine (UDP-GlcNAc) in Pt. In addition, Pt tissue was distinguished from LG tissue by differences in 20 assigned metabolites (and an additional six unassigned resonances) (Table 2 and Supplementary Figures S1C,D), with 15 of the identified metabolites remaining significantly different upon FDR correction. The differences in alanine, GPC, NAD^+^, phenylacetate and succinate were not significant, after FDR correction (^c^ in Table 2).

**TABLE 2:** Polar pancreatic metabolites varying between pancreatitis induced-mice (Pt) and normal tissue (N) (left), and between Pt and LG PanIN lesions (LG) (right).

Metabolite	δ/ppm (multiplicity)^a^	Pt (*n* = 6) vs*.* N (*n* = 7)				Pt (*n* = 6) vs*.* LG (*n* = 5)			
		Tissue signature		Cell signature		Tissue signature		Cell signature	
		ES ± error	*p*-value	ES ± error	*p*-value	ES ± error	*p*-value	ES ± error	*p*-value
3-HIVA	1.25 (s)	—	—	−1.7 ± 1.3^b^	1.2E−3	−1.7 ± 1.4	4.3E−3	—	—
ADP ^c^	8.28 (s)	3.5 ± 1.7	2.9E−3	—	—	3.3 ± 1.8^b^	4.3E−3	2.2 ± 1.5	4.3E−3
Alanine ^c^	1.48 (d)	−5.8 ± 2.5^b^	3.9E−5	−5.3 ± 2.3	2.4E−5	−2.1 ± 1.5^b^ ^,^ ^d^	4.0E−2	−1.8 ± 1.4^d^	3.2E−2
AMP ^c^	8.60 (s)	−4.5 ± 2.1^b^	1.1E−5	−4.9 ± 2.2	7.5E−6	−7.4 ± 3.3^b^	2.1E−6	−3.2 ± 1.8	5.1E−3
ATP ^c^	6.14 (d)	5.2 ± 2.3	2.5E−5	—	—	6.2 ± 2.9^b^	5.9E−6	1.7 ± 1.4^d^	3.1E−2
Creatine	3.04(s)	−4.4 ± 2.0^b^	1.7E−4	−3.7 ± 1.8	3.1E−4	—	—	—	—
Creatinine	3.05 (s)	—	—	−2.1 ± 1.4^b^	4.6E−3	4.0 ± 2.0	4.3E−3	—	—
Formate ^c^	8.46 (s)	−3.8 ± 1.8	1.2E−3	−6.6 ± 2.8	5.7E−7	−4.3 ± 2.1^b^	4.3E−3	−2.4 ± 1.6^d^	8.8E−3
Fumarate	6.52 (s)	−1.7 ± 1.3^b^	1.5E−2	−3.8 ± 1.8	1.3E−4	—	—	−2.3 ± 1.5^d^	3.1E−2
Glucose	5.23 (d)	2.2 ± 1.3	2.2E−3	—	—	—	—	−1.9 ± 1.4^d^	4.9E−2
Glutamate	2.35 (m)	5.1 ± 2.3	7.2E−6	−1.6 ± 1.2^d^	4.8E−2	—	—	—	—
Glutamine	2.45 (m)	—	—	−2.5 ± 1.5^b^	3.6E−3	3.2 ± 1.8	1.2E−3	—	—
GPC ^c^	3.23 (s)	−1.4 ± 1.2^b^ ^,^ ^d^	4.3E−2	−2.7 ± 1.5	1.0E−3	−2.0 ± 1.5^b^ ^,^ ^d^	4.6E−2	−2.0 ± 1.4^d^	2.1E−2
GSH ^c^	4.57 (m)	9.5 ± 3.8	1.2E−3	—	—	12 ± 5.3	4.3E−3	1.7 ± 1.4^d^	3.1E−2
Histidine	7.08 (s)	−2.7 ± 1.5^b^	9.2E−4	−4.9 ± 2.2	9.0E−5	—	—	—	—
Lactate	1.33 (d)	—	—	−2.0 ± 1.3^b^	9.3E−3	−2.7 ± 1.7^b^	1.8E−2	−2.2 ± 1.5^d^	2.8E−2
Leucine	0.96 (t)	—	—	−1.7 ± 1.3^b^	1.8E−2	1.9 ± 1.4	1.9E−2	—	—
NAD^+^	9.34 (s)	—	—	−1.7 ± 1.3^b^	1.7E−2	2.0 ± 1.4^d^	3.9E−2	—	—
Niacinamide ^c^	8.94 (d)	−2.9 ± 1.6^b^	7.6E−4	−3.2 ± 1.7	5.1E−4	−3.5 ± 1.9^b^	3.6E−3	−2.2 ± 1.5^d^	3.1E−2
PC	3.22(s),4.17 (m)	−2.3 ± 1.4^b^	2.8E−3	−3.6 ± 1.8	1.0E−4	3.8 ± 2.0	1.7E−3	—	—
Phenylacetate ^c^	7.26 (t)	3.4 ± 1.7	2.9E−3	—	—	2.0 ± 1.5^d^	2.9E−2	—	—
Phenylalanine	7.42 (t)	4.0 ± 1.9	4.4E−5	—	—	—	—	—	—
Succinate	2.41 (s)	—	—	−2.5 ± 1.4^b^	1.9E−3	−2.2 ± 1.5^d^	2.9E−2	—	—
Taurine	3.26 (t)	2.8 ± 1.5	1.2E−3	—	—	—	—	—	—
TMAO ^c^	3.27 (s)	−2.0 ± 1.3^b^	8.6E−3	−4.0 ± 1.9	4.6E−5	−1.6 ± 1.4	4.3E−3	—	—
Tyrosine	7.20 (d)	−3.4 ± 1.7^b^	3.7E−4	−3.5 ± 1.7	1.7E−4	—	—	—	—
UDP-Glc/GlcA	7.95 (d)	—	—	−2.7 ± 1.5^b^	1.5E−3	4.1 ± 2.1	7.4E−4	—	—
UDP-GlcNAc	5.52 (dd)	−3.3 ± 1.7^b^	6.2E−4	−4.8 ± 2.1	1.8E−5	—	—	—	—
UMP ^c^	8.10 (d)	−2.1 ± 1.4^b^	4.5E−4	−3.9 ± 1.8	6.8E−5	−3.3 ± 1.8^b^	3.0E−3	−1.9 ± 1.4^d^	2.9E−2
Valine	1.05 (d)	−2.0 ± 1.3^b^	5.9E−3	−3.7 ± 1.8	5.7E−5	—	—	—	—
Unassigned resonances									
U1 δ1.58	1.58 (d)	−1.7 ± 1.3^b^	2.2E−2	−2.1 ± 1.4	1.2E−3	—	—	—	—
U2 δ2.08	2.08 (s)	−2.4 ± 1.4^b^	4.0E−3	−3.2 ± 1.7	2.1E−4	—	—	—	—
U3 δ2.68	2.68 (t)	3.3 ± 1.7^d^	1.9E−4	—	—	—	—	—	—
U4 δ5.16 (saccharide) ^c^	5.16 (d)	10.4 ± 4.1	4.8E−9	—	—	13 ± 5.6^b^	4.3E−3	2.2 ± 1.5	1.3E−7
U5 δ 5.56	5.56 (dd)	—	—	−4.2 ± 1.9^d^	6.5E−5	—	—	—	—
U6 δ5.70 ^c^	5.70 (s)	3.1 ± 1.6	1.3E−3	—	—	3.8 ± 2.0^b^	9.5E−4	3.8 ± 2.0	9.5E−4
U7 δ6.03 ^c^	6.03 (d)	1.6 ± 1.3	2.5E−2	—	—	7.1 ± 3.2	5.3E−4	—	—
U8 δ6.80	6.80 (s)	−1.6 ± 1.2^b^	2.5E−2	−1.6 ± 1.2	2.9E−2	3.5 ± 1.9^b^	6.7E−3	−3.1 ± 1.8	4.3E−3
U9 δ 7.49	7.49 (t)	—	—	−3.0 ± 1.6^d^	1.3E−3	−3.7 ± 2.0^d^	4.8E−4	−2.4 ± 1.6	1.6E−2
U10 δ8.21 ^c^	8.21 (s)	−1.7 ± 1.3^b^	2.1E−2	−2.8 ± 1.5	1.3E−3	−2.4 ± 1.6^d^	3.1E−2	—	—

Effect sizes (ES) were calculated using normalization to total area (Tissue metabolic signature) and to average cell numbers (Cell metabolic signature). Only variations with ES higher than the error and *p*-value < 0.05 are shown.

a Chemical shifts of integrated peaks, within each spin system.

b Variation maintained (in direction and approximate magnitude, considering the ES error) or becoming detectable in cell signature, thus proposed to reflect cellular deviant metabolism.

c Metabolites comprised in the proposed Pt-specific metabolic signature (also underlined); s, singlet; d, doublet; dd, doublet of doublets; t, triplet; m, multiplet; 3-HIVA, 3-hydroxyisovalerate; ADP, adenosine diphosphate; AMP, adenosine monophosphate; ATP, adenosine triphosphate; GPC, glycerophosphocholine; GSH, glutathione (reduced); NAD+, nicotinamide adenine dinucleotide (oxidized); PC, phosphocholine; TMAO, trimethylamine N-oxide; UDP-Glc/GlcA, uridine diphosphate glucose/glucoronate; UDP-GlcNAc, uridine diphosphate-N-acetylglucosamine; UMP, uridine monophosphate; Ui, unassigned resonance.

d *p*-value > 0.05 after FDR correction.

**FIGURE 3:**
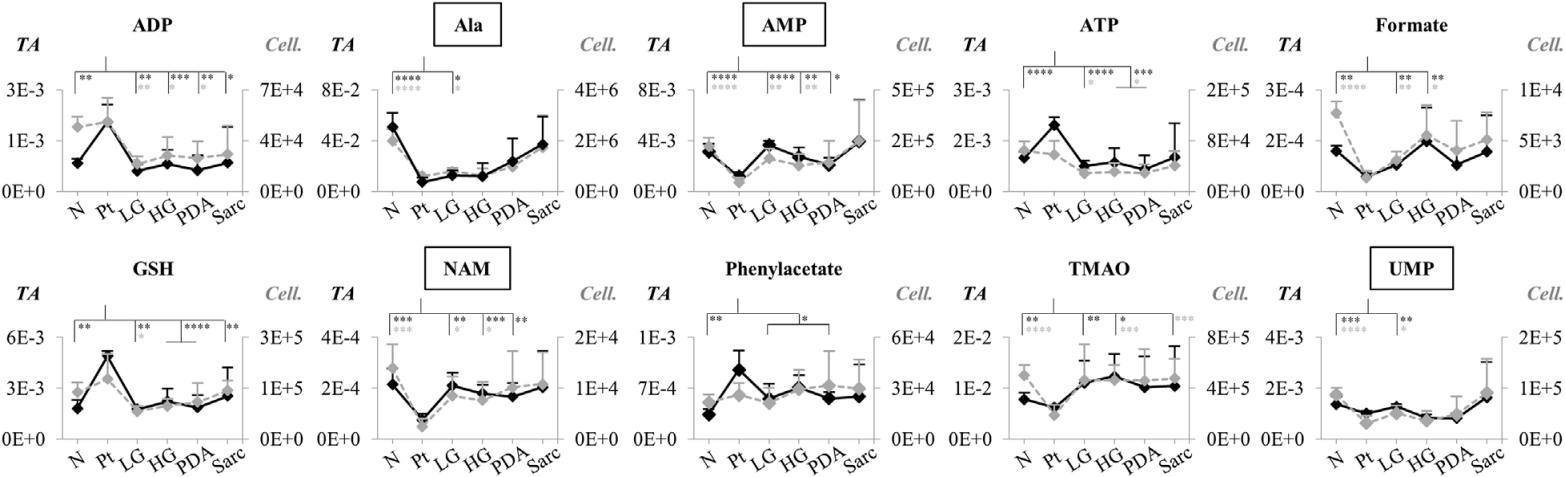
Variations in 10 metabolites distinguishing Pt tissue from both N and LG tissues (GPC variations are left out as they become non-significant upon FDR correction, ^d^ in Table 2), represented as normalized peak integrals, either in relation to total spectra area (TA) (*Tissue signature:* left axes, filled black lines) or cellularity (*Cell signature:* right axes, dashed grey lines). Asterisks indicate statistical relevance of pairwise comparisons in relation to Pt (black and grey asterisks correspond to *TA* and *Cell.* data, respectively): ∗*p*-value < 0.05, ∗∗*p*-value < 0.01, ∗∗∗*p*-value < 0.001, ∗∗∗∗*p*-value < 0.0001. Boxed metabolite names refer to those suggested to reflect intracellular metabolic variations (similar black and grey lines, for the N, Pt and LG groups). Abbreviations as defined in caption of Figure 1 and Supplementary Table S1.

For those metabolites expected to be exclusively intracellular (ATP, ADP, GSH, AMP, UDP-GlcNAc), a correction was necessary to account for the higher tissue cellularity that characterized Pt tissue as shown by previous histological analysis (Serrao et al., 2016). Hence, spectra were normalized according to the number of cells instead of by total spectral area. This correction was applied to all those metabolites comprising the tissue signature and allowed an approximate assessment of changes in their intracellular concentrations. Therefore, we defined a Tissue metabolic signature and a Cellular metabolic signature when comparing Pt with both N and LG samples (Table 2). Following this correction, there was no change in the intracellular concentrations of ATP, ADP, GSH, glucose, phenylacetate, phenylalanine and taurine between Pt and N samples (*Cell signature* in Table 2). In the Cell signature of Pt vs*.* N (^b^ in Table 2), changes remained in the concentrations of AMP and UDP-GlcNAc and another 12 metabolites, whereas new changes were revealed for 3-hydroxyisovalerate (3-HIVA), creatine, glutamine, lactate, leucine, NAD^+^, succinate and UDP-Glucose/Glucuronate (UDP-Glc/GlcA). A similar approach was applied to Pt vs*.* LG, producing a Cell signature (Table 2) which, upon FDR correction, comprised changes in ADP and AMP concentrations and smaller changes in 10 other metabolites (^c^ in Table 2). Table 2 shows that a common set of changes in 10 metabolites (excluding GPC, which was only slightly decreased, becoming non-significant upon FDR) characterize Pt tissue, whether compared to the N or LG groups (^d^ in Table 2, under *Tissue signature*). This Pt tissue signature included increased concentrations of ADP, ATP, GSH, phenylacetate, and decreased concentrations of alanine, AMP, formate, NAM, trimethylamine *N*-oxide (TMAO) and UMP (Figure 3, black lines relating to N, Pt and LG tissue groups). We suggest that this 10-metabolite signature may be capable of differentiating Pt tissues from both N and LG tissues. Within the above changes, the decreases in alanine, AMP, NAM and UMP (boxed metabolite names in Figure 3, ^b^ in Table 2) are independent of cellularity (note the overlap between black and grey lines in Figure 3). The remaining changes (ADP, ATP, formate, GSH and phenylacetate) arise, at least in part due to the higher cell density characterizing Pt tissue, but nevertheless form part of the overall tissue signature. The dependence of TMAO concentrations on tissue cellularity is unclear, as it features in the cell signature of Pt vs*.* N, but not in that of the Pt vs*.* LG comparison.

### Metabolic profiling of pancreatic cancer progression

**FIGURE 4:**
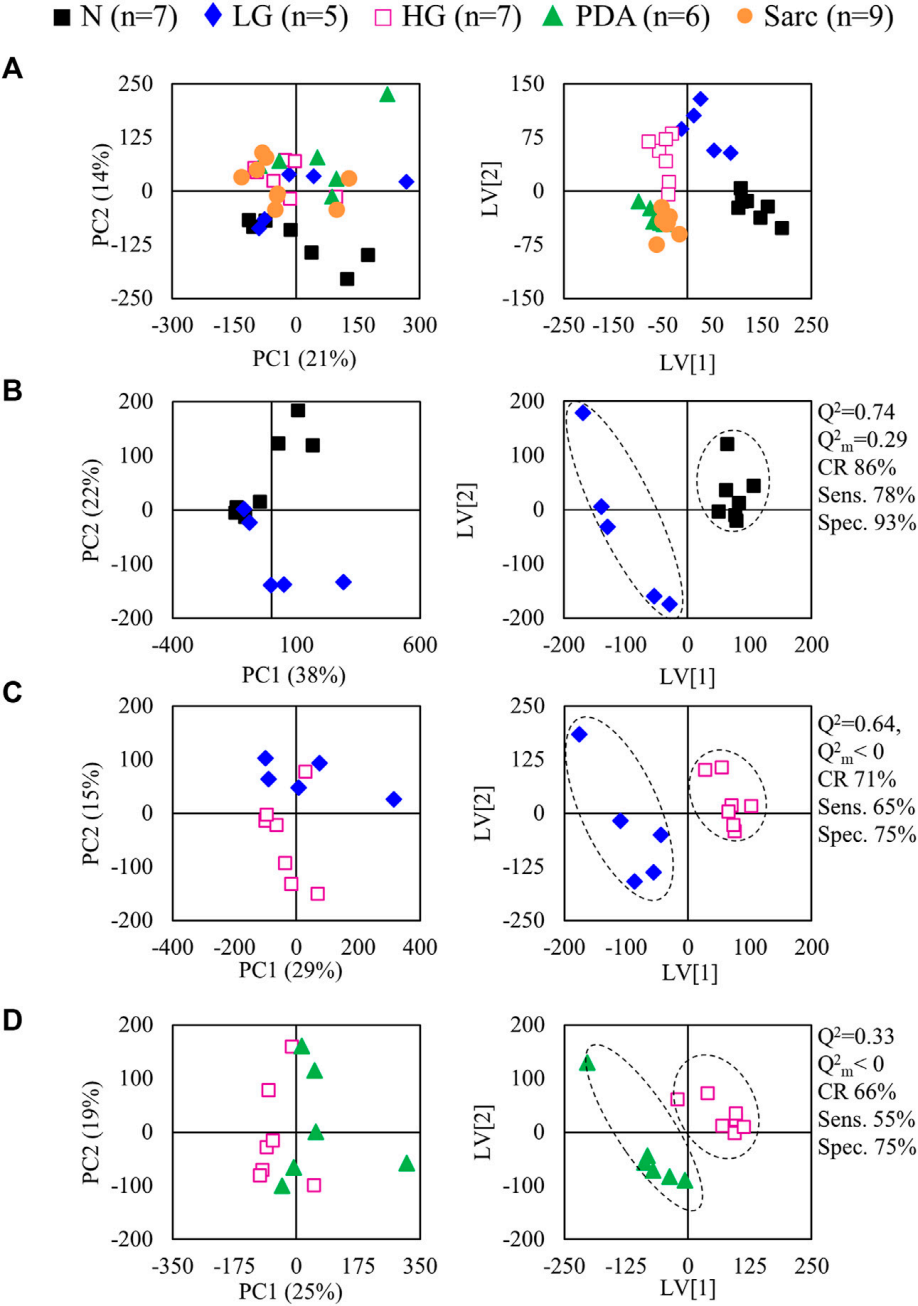
PCA (left) and PLS-DA (right) scores scatter plots for ^1^H NMR spectra (normalized to total area) of **(A)** N, LG, HG, PSA and Sarc. classes, **(B)** N vs*.* LG, **(C)** LG vs*.* HG, **(D)** HG vs*.* PDA. Q^2^, predictive power of PLS-DA model; CR, classification rate; sens., sensitivity; spec., specificity.

Application of PCA to ^1^H NMR spectra obtained from N, LG, HG, PDA and Sarc pancreatic tissue, where the signal intensities were normalized to total spectral area, showed some separation of N samples from the later disease stages (Figure 4A). This was improved by PLS-DA (Figure 4B). PCA and PLS-DA analyses of pairwise comparisons between successive stages of the disease also showed some separation (Figures 4B,C), although with no/low statistical robustness for classification (as shown by MCCV results, Supplementary Table S2), which is due mainly to the gradual stepwise variations of most metabolites. Here, cell numbers were similar between the groups (Table 1), so only a Tissue Signature (total area normalization) was considered. LG lesions were distinguished from N tissue by changes in the concentrations of 15 identified metabolites (Table 3), with glucose and histidine showing less significant changes (^b^ in Table 3). In LG tissue, robust changes when compared to N tissue comprised decreases in alanine, creatine, lysine, phosphocholine (PC), tyrosine, UDP-Glc/GlcA, UDP-GlcNAc and valine, while increases were noted for aspartate, glutamate, *m-*inositol, phenylalanine and sucrose. Progression from LG to HG was characterized by significantly decreased concentrations (in HG) of 3-methylxanthine, glutamate and UMP, in addition to smaller changes affecting nine other metabolites (increased glucose, and decreased 2-phosphoglycerate, alanine, aspartate, *m-*inositol, PC, sucrose and UDP-Glc/GlcA (Table 3). PDA samples were distinguished from HG by significant decreases in glucose and phosphoethanolamine (PE) and an increase in proline concentration, together with smaller increases in 3-hydroxybutyrate (3-HBA), 3-hydroxyisobutyrate (3-HIBA), alanine and lactate, and a small decrease in ascorbate concentration. Regarding the comparison between PDA and Sarc samples, only a small relative increase in UDP-Glc/GlcA was observed in the Sarc samples. However, in assessing disease progression mouse ages differed, particularly for the HG (9 months) and PDA (3–6 months) groups, compared to the remaining groups (1.5–4 months). Mouse aging over considerably longer periods has been reported to impact the metabolite profile of skeletal muscle up to 28 months (Uchitomi et al., 2019), and that of plasma over 18 months (Seo et al., 2016). Considering those metabolites that have been reported to be age-dependent, and despite the large age differences compared with the mice studied here, the levels of *m-*inositol, PC and proline (for the PDA and HG groups) may potentially include contributions due to mouse aging (^c,d^ in Table 3).

**TABLE 3:** Polar pancreatic metabolites varying across progression from normal pancreatic tissue (N), through LG PanIN lesions, HG PanIN lesions, to PDA and sarcomatoid carcinoma (Sarc).

Metabolite	δ/ppm (multiplicity)^a^	LG (*n* = 5) vs*.* N (*n* = 7)		HG (*n* = 7) vs*.* LG (*n* = 5)		PDA (*n* = 6) vs*.* HG (*n* = 7)		Sarc (*n* = 9) vs*.* PDA (*n* = 6)	
		ES ± error	*p*-value	ES ± error	*p*-value	ES ± error	*p*-value	ES ± error	*p*-value
2-Phosphoglycerate	4.43 (m)	—	—	−1.6 ± 1.3^b^	2.8E−2	—	—	—	—
3-HBA	1.20 (d)	—	—	—	—	1.5 ± 1.2^b^	5.0E−2	—	—
3-HIBA	1.08 (d)	—		—	—	1.4 ± 1.2^b^	2.2E−2	—	—
3-Methylxanthine	8.03 (s)	—	—	−2.9 ± 1.6	5.1E−3	—	—	—	—
Alanine	1.48 (d)	−4.6 ± 2.2	2.5E−5	—	—	1.4 ± 1.2^b^	3.8E−2	—	—
AMP	8.60 (s)	—	—	−1.5 ± 1.3^b^	2.5E−2	—	—	—	—
Ascorbate	4.51 (d)	—	—	—	—	−1.4 ± 1.2^b^	4.2E−2	—	—
Aspartate	2.80 (d)	3.6 ± 1.8	2.8E−3	−2.2 ± 1.5^b^	3.1E−2	—	—	—	—
Creatine	3.04 (s)	−3.6 ± 1.8	1.3E−4	—	—	—	—	—	—
Glucose	5.23 (d)	2.3 ± 1.5^b^	4.1E−2	1.6 ± 1.3^b^	3.9E−2	−3.6 ± 1.8	2.3E−3	—	—
Glutamate	2.35 (m)	5.0 ± 2.3	7.3E−4	−2.8 ± 1.6	1.9E−3	—	—	—	—
GPC	4.33 (m)	—	—	−1.9 ± 1.4	3.7E−2	—	—	—	—
Histidine	7.05 (s)	−2.1 ± 1.4^b^	3.1E−2	—	—	—	—	—	—
Lactate	1.33 (d)	—	—	—	—	1.9 ± 1.3^b^	1.7E−2	—	—
Lysine	1.70 (m)	−1.8 ± 1.3	2.5E−2	—	—	—	—	—	—
*m*-inositol	4.06 (t)	2.3 ± 1.5	2.6E−2	1.8 ± 1.4^b^ ^,^ ^c^	4.6E−2	—	—	—	—
PC	4.17 (m)	−4.4 ± 2.1	3.7E−4	−3.1 ± 1.7^b^ ^,^ ^c^	8.1E−3	—	—	—	—
PE	3.98 (m)	—	—	—	—	−2.6 ± 1.5	1.2E−3	—	—
Phenylalanine	7.42 (t)	2.3 ± 1.5	2.4E−2	—	—	—	—	—	—
Proline	1.99 (m)	—	—	—	—	1.8 ± 1.2^d^	1.4E−2	—	—
Sucrose^e^	5.42 (d)	1.9 ± 1.4	1.8E−2	1.8 ± 1.4^b^	2.1E−2	—	—	—	—
Tyrosine	7.20 (d)	−3.3 ± 1.7	2.4E−4	—	—	—	—	—	—
UDP-Glc/GlcA	7.95 (d)	−2.9 ± 1.6	3.3E−3	−2.1 ± 1.4^b^	1.8E−2	—	—	1.4 ± 1.2^b^	1.2E−2
UDP-GlcNAc	5.52 (dd)	−3.6 ± 1.9	4.2E−3	—	—	—	—	—	—
UMP	8.10 (d)	—	—	−3.5 ± 1.8	1.5E−4	—	—	—	—
Valine	1.05 (d)	−2.1 ± 1.4	7.4E−3	—	—	—	—	—	—
U2 δ2.08	2.08 (s)	−2.3 ± 1.5	3.2E−3	—	—	—	—	—	—
U3 δ2.68	2.68 (t)	3.3 ± 1.7	4.9E−4	−5.1 ± 2.3	2.3E−5	—	—	—	—
U4 δ5.16 (saccharide)	5.16 (d)	—	—	−2.4 ± 1.5^b^	1.8E−2	—	—	—	—
U5 δ5.56	5.56 (dd)	−1.9 ± 1.4	2.4E−2	—	—	—	—	—	—
U9 δ7.49	7.49 (t)	4.0 ± 2.0	1.5E−4	—	—	—	—	—	—
U11 δ2.59	2.59 (s)	1.8 ± 1.3	1.5E−2	—	—	—	—	—	—

Effect sizes (ES) were calculated using normalization to total area (*tissue metabolic signature*) only, as cell numbers are broadly similar for these groups. Only variations with ES higher than the error and *p*-value < 0.05 are shown. s, singlet; d, doublet; dd, doublet of doublets; t, triplet; m, multiplet. 3-HBA, 3-hydroxybutyrate; 3-HIBA, 3-hydroxyisobutyrate; PE, phosphoethanolamine; other compound abbreviations as defined in Table 2 and Supplementary Table S1. Ui, unassigned resonance.

a Chemical shifts of integrated peaks.

b *p*-value > 0.05 after FDR correction.

c Metabolite changes with possible contribution from effects of mice age according to Uchitomi et al. (2019).

d Metabolite changes with possible contribution from effects of mice age according to Seo et al. (2016).

e Possibly of a dietary origin.

**FIGURE 5:**
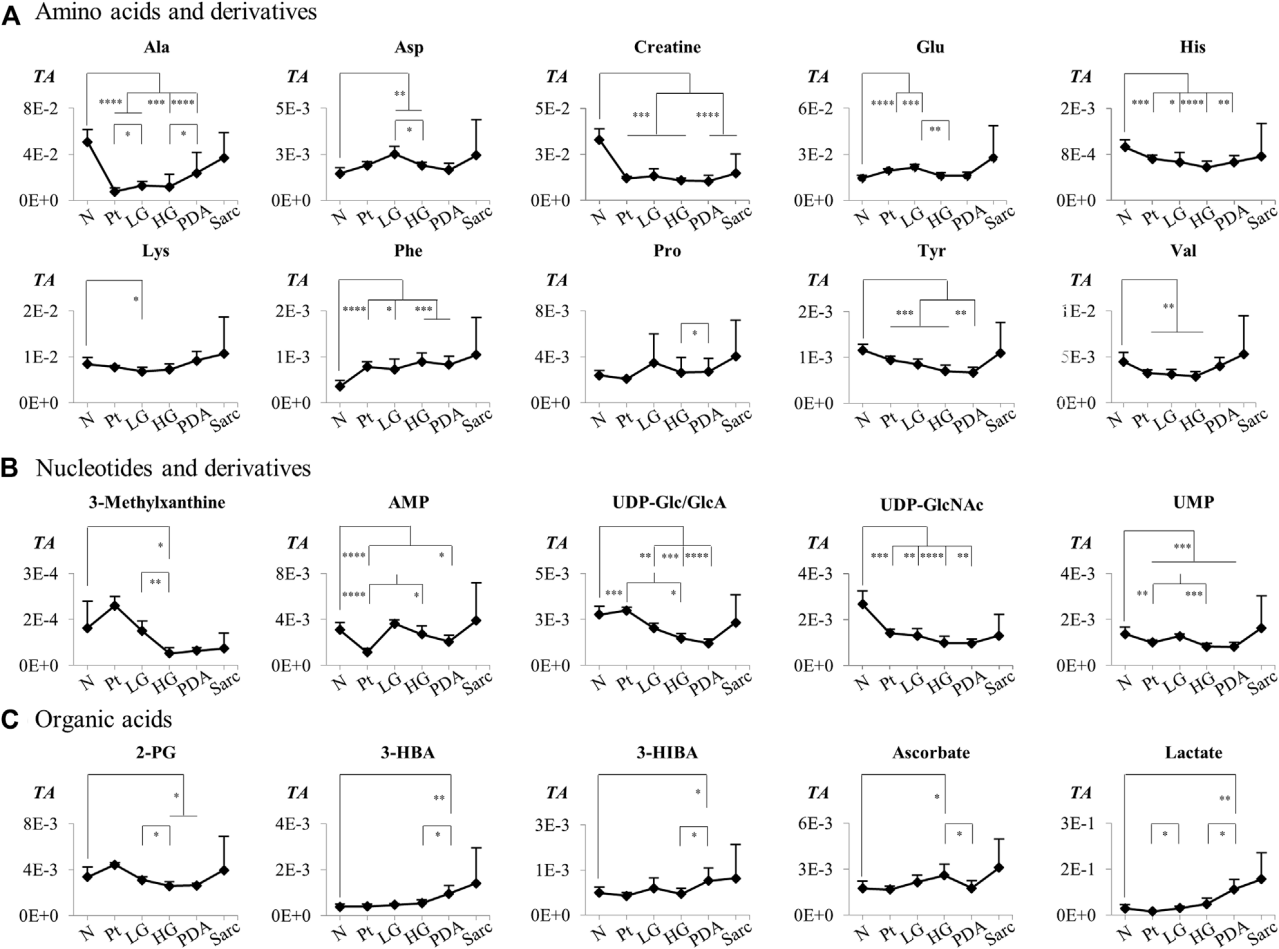
Variations in **(A)** amino acids and derivatives, **(B)** nucleotides and related compounds, and **(C)** organic acids across pancreatic cancer progression, represented as normalized peak integrals in relation to total spectra area (TA). Abbreviations: 2-PG, 2-phosphoglycerate; Three-letter code used for amino acids and other abbreviations as defined in caption of Figure 1 and Supplementary Table S1. Asterisks indicate statistical relevance of pairwise comparisons in relation to Normal tissue (N): ∗*p*-value < 0.05, ∗∗*p*-value < 0.01, ∗∗∗*p*-value < 0.001, ∗∗∗∗*p*-value < 0.0001.

**FIGURE 6:**
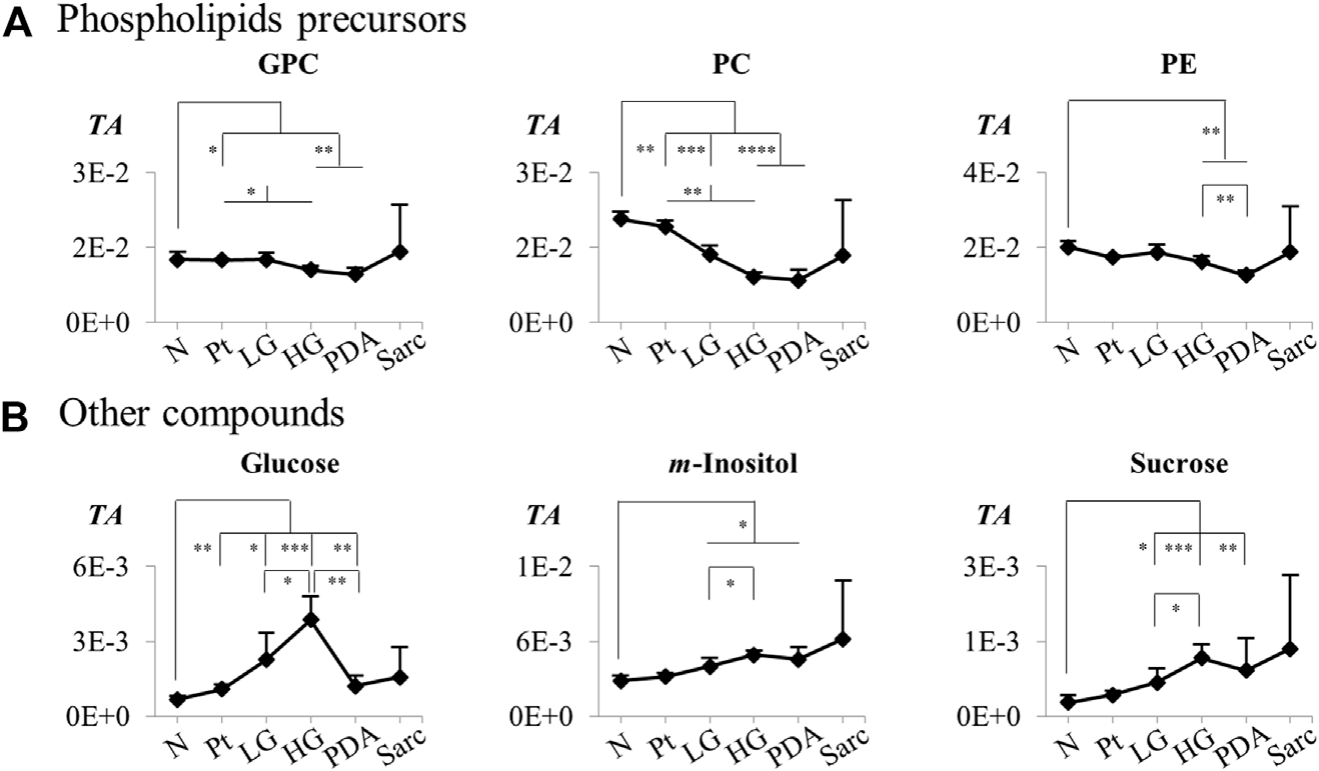
Variations in **(A)** phospholipids precursors and **(B)** other compounds across pancreatic cancer progression, represented as normalized peak integrals in relation to total spectra area (TA). Abbreviations as defined in caption of Figure 1 and Supplementary Table S1. Asterisks indicate statistical relevance of pairwise comparisons in relation to Normal tissue (N): ∗*p*-value < 0.05, ∗∗*p*-value < 0.01, ∗∗∗*p*-value < 0.001, ∗∗∗∗*p*-value < 0.0001.

The individual metabolites (Figures 5, 6) showed gradual changes in concentration with disease progression (N-LG-HG-PDA/Sarc). These changes included a decrease in alanine concentration from N to LG, followed by an increasing concentration that differentiates PDA from HG, and increases in lactate concentration with disease progression, particularly from HG to PDA (Table 3 and Figure 5). These are changes that have been observed previously in this disease model (Serrao et al., 2016). The metabolite concentration changes that can potentially differentiate cancer stages LG and PDA include: *1*) alanine, lysine and valine (increasing); and tyrosine, glutamate and aspartate (decreasing) (Figure 5A); *2*) nucleotides and glycosylated UDP derivatives (decreasing) (Figure 5B); *3*) lactate and the ketone body 3-HBA (increasing) (Figure 5C); and *4*) membrane precursors PC, PE and to a lesser extent, GPC (decreasing) (Figure 6A). Glucose levels suggest an increasing concentration up to LG, followed by a marked decrease between HG and PDA (Figure 6B).

## Discussion

### Metabolic signature of induced acute Pt

Previous metabolomic studies in animal models of Pt have mostly characterized the metabolic changes associated with Pt, when compared to healthy controls (Peng et al., 2021) or to established PCa (Mehta et al., 2017). An early study showed increased lipid content and decreased PC and GPC levels in PDA, compared to chronic Pt (Fang et al., 2007). Studies of progressive chronic Pt in swine (Sun et al., 2014) and rat (Tian et al., 2015) models showed gradual decreases in PC, GPC, betaine and glycine in the swine model, and changes in aspartate, betaine and lipids, which in the rat model correlated with the degree of fibrosis and/or inflammatory cell infiltration. GC-MS profiling of tissue extracts and serum (Sakai et al., 2012) in mice with acute induced Pt showed significant changes in tricarboxylic acid (TCA) cycle intermediates, glutamate and *O-*phosphoethanolamine, whereas serum profiling using Ultra Performance Liquid Chromatography (UPLC)-MS suggested that a 19-metabolite signature could distinguish animals with acute Pt from controls (Guo et al., 2019). Recent studies in human subjects have also reported potential blood markers that could distinguish chronic pancreatitis from PDA (Lindahl et al., 2017; Mayerle et al., 2018). Regarding caerulein-induced acute Pt, this work complements a recent targeted UPLC-MS/MS metabolomics study of the same animal model (in tandem with proteomics), which aimed to characterize the changes in the methionine cycle and transsulfuration pathway, which are believed to become dysregulated due to the inflammatory process accompanying acute Pt (Rius-Pérez et al., 2020). Here, we report a full metabolic signature of the pancreata of mice affected by induced acute Pt and compare it to both normal tissue and precancerous tissue, in search of a specific metabolic signature that may potentially be used as a clinical marker of Pt.

Our data has shown that account should be taken of the relatively higher cellularity of Pt tissue, before results may be interpreted as due to changes in intracellular concentrations. This was the case for ATP, ADP, GSH, glucose, phenylacetate, phenylalanine and taurine, the levels of which were elevated compared to normal tissue, which may be due to the higher cell density in Pt tissue. Independently of cellularity, we have found a 22-metabolite signature of Pt that, compared to N tissue, comprised decreases in the amino acids alanine, glutamate, glutamine, histidine, and tyrosine, which have been reported previously as being related to their anaplerotic role in increasing TCA cycle activity to meet the increased energy demand in Pt tissue (Sakai et al., 2012). We observed additional decreases in leucine and valine, which were reported previously to change between acute and chronic Pt (Ma et al., 2012) and which are also consistent with an anaplerotic role. Enhanced TCA cycle activity is further supported by decreased levels of TCA cycle intermediates succinate and fumarate in Pt tissue, in broad agreement with previous reports indicating increases and decreases of TCA cycle intermediates at the beginning and end of the cycle, respectively (Sakai et al., 2012). Variations in creatine and creatinine may also be indicative of a disturbance in energy metabolism, although creatinine is also considered as a marker of pancreatic necrosis in acute Pt (Lankisch et al., 2010)^.^ The decreases in membrane biosynthetic precursors (GPC, PC and PE) agree, in part, with lower GPC and/or PC levels reported for progressive chronic Pt in pigs (Sun et al., 2014) but appear to contradict increased choline compounds observed in Wistar rats affected by either acute or chronic Pt, as determined by high resolution magic angle spinning NMR, and interpreted as indicative of higher rates of cell proliferation (Ma et al., 2012). In the same study, lactate was decreased in Pt tissue, in agreement with our observations in the cell signature of Pt vs*.* N. However, regarding both studies mentioned above (Ma et al., 2012; Sun et al., 2014), it is unclear if the metabolic features of different animal models may be directly comparable. The decreased levels of NAD^+^ and NAM observed here agree with a recent study relating such variations with macrophage activation and inflammation (He et al., 2021). Here we found lower concentrations of UMP in acute Pt, while increased UTP has been reported and interpreted as accompanying higher cell proliferation rates (Choi et al., 2013). The decrease in UMP concentration may be the result of its conversion to UDP and subsequently to UDP-Glc/GlcA and UDP-GlcNAc, which are required in Pt tissue to support increased posttranslational protein *O-*glycosylation (Ryczko et al., 2016). The significant decrease in TMAO may reflect its role as a mediator of inflammatory processes (Janeiro et al., 2018), deriving from the oxidation of trimethylamine (TMA) generated *via* gut microbial metabolism of choline, betaine (reported to increase in chronic Pt (Tian et al., 2015)) and carnitine. We could not confirm the changes in betaine and aspartate concentrations reported previously for chronic Pt, in tandem with increased fatty acids levels (Tian et al., 2015), which suggests that acute and chronic Pt may be characterized by different metabolic signatures, at least in part.

The comparison of Pt with LG tissue exhibited generally smaller, but still statistically significant, changes (compared to Pt vs*.* N) both in the tissue and cell signatures. In the cell signature of precancerous tissue, the higher levels of alanine, AMP and lactate and lower levels of ATP, ADP and GSH may reflect increased energy demand and mobilization of anti-oxidative defenses in the precancerous tissue.

The differentiation of low-grade disease (LG) from both Pt or N tissue is of potential clinical relevance. Notably, when compared to both N and LG tissues, the pancreatic tissue of mice with Pt exhibits a common set of changes, which we propose to be specific to Pt tissue, namely: increased ADP, ATP, GSH, phenylacetate, and decreased alanine, AMP, formate, GPC, NAM, TMAO, and UMP. If confirmed in human studies, this 10-metabolite signature may potentially serve as a marker of pancreatic tissue affected by acute Pt. This may help to confirm histological differentiation of this benign condition from both control and precancerous conditions, for instance if magnetic resonance spectroscopy is coupled with the current magnetic resonance cholangiopancreatography (MRCP) methods (Shah et al., 2018).

### Metabolic signature of pancreatic cancer progression

In LG tissue (composed of PanIN 1 and 1A lesions), compared to N tissue, metabolite concentration changes were observed for: *1*) amino acids, with decreases in alanine, creatine, lysine, tyrosine and valine; and increases in aspartate, glutamate and phenylalanine; *2*) an increase in *m-*inositol and decreases in PC, UDP-Glc/GlcA and UDP-GlcNAc (Figures 5, 6). Smaller changes were noted for glucose and histidine, and no evidence of an early increase in lactate concentration was noted between N and LG samples. A decrease in alanine concentration between normal tissue and LG samples has been observed previously in the Kras model (Serrao et al., 2016), this amino acid having been identified as preferentially being up taken by tumor cells to feed the TCA cycle, to support the biosynthesis of other non-essential amino acids and lipids (Dutta et al., 2019; Pupo et al., 2019). The specific role of alanine in PDA has been recognized as a sign that a metabolic shift occurs to decrease tumor dependence on glucose and glutamine-derived carbon and enhance the use of alanine as an alternative carbon source. From LG to PDA, we observed a small increase in alanine concentration, which may reflect increased alanine production by pancreatic stellate cells (Sousa et al., 2016; Pupo et al., 2019). Valine was the only branched-chain amino acid (BCAA) observed to vary, decreasing from N to LG, which is contrary to a metabolomics study of a different model of PanIN (comprising PanIN 1, 2 and 3) and PDA obtained by injecting 7,12-dimethylbenzanthracene (7,12-DMBA) into the pancreas of Sprague-Dawley (SD) rats (Wen et al., 2016). In this model the three BCAAs were elevated in PanIN pancreatic extracts compared to controls. In the Kras mouse model used here it is possible that the decrease in valine concentration between N and LG may reflect its enhanced use in anaplerosis. This would be consistent with the observed increases in plasma circulating BCAAs reported in the early stages of PCa in mice and in humans, which is probably due to protein catabolism to supply anaplerotic substrates (Mayers et al., 2014; Xu et al., 2020). The valine concentrations measured here showed a non-significant increase in the later disease stages, which was also noted previously for 15- and 25-week old KPC mice, affected by PanIN lesions and PDA, respectively (Vernucci et al., 2019) Lysine and tyrosine are both ketogenic amino acids, that produce acetyl CoA and acetoacetate, respectively, with tyrosine also potentially acting as a gluconeogenic amino acid, entering the TCA cycle *via* fumarate. The decreases in these amino acids between N and LG are consistent with an enhancement of TCA cycle activity. During disease progression, lysine and tyrosine concentrations tended to increase and decrease, respectively. These changes agree with their proposed respective upregulation and down regulation reported for 25-week-old KPC mice, bearing PDA (Vernucci et al., 2019).

The decrease in creatine, which was also observed in the pancreas of SD rats with PanIN lesions (Wen et al., 2016), may reflect increased conversion to phosphocreatine, which buffers the ATP concentration (Papalazarou et al., 2020). Creatine concentrations have been shown to be inversely related to tumor volume in pancreatic cancer patients (Chang et al., 2021). Increased glutamate, largely arising from enhanced glutaminolysis, is used by Kras transformed cells for anaplerosis and nucleotide production (Pupo et al., 2019). In PDA cells, glutamate is used by mitochondrial aspartate transaminase GOT2 to synthesize aspartate and α-ketoglutarate. Here we observed increases in glutamate and aspartate concentrations in LG when compared to N. Aspartate is eventually converted into malate and pyruvate *via* aspartate transaminase GOT1 and contributes to an increased NADPH/NADP^+^ ratio and maintenance of cell redox potential (Mohelnikova-Duchonova et al., 2013). Phenylalanine and *m*-inositol, a membrane component and osmolyte (Majumder and Biswas, 2006), were also observed to increase in PanIN tissue of an DMBA-induced SD rat model, compared to normal tissue (Wen et al., 2016). An increase in phenylalanine, as seen here between N and LG and, to a lesser extent, throughout disease progression, may be indicative of inflammation and immune activation (Neurauter et al., 2008).

PC levels decreased between N and LG samples, and thereafter during disease progression, suggesting an enhanced demand for membrane precursors to sustain cell proliferation. Although there is a tendency for the concentration of choline compounds to increase in cancer cells, including in PDA (Penet et al., 2015; Saito et al., 2022), our results are in agreement with a previous report comparing PDA with chronic Pt, which indicated decreases in PC in PDA (Fang et al., 2007). It was suggested that the decrease in PC concentration may result from inhibition of Cho-kinase and PC transferase or consumption of PC through the CDP-Cho pathway. Our observation of strongly decreased concentrations of glycosylated uridine compounds between N and LG is consistent with decreased uridine and UDP levels in PanIN2 tissue in a SD rat model of PanIN and PDA (Wen et al., 2016). This may be explained by upregulation of the hexosamine biosynthesis pathway (HBP) by oncogenic Kras, which leads to the production of UDP-GlcNAc and other nucleotide hexosamines (Sousa and Kimmelman, 2014; Yan et al., 2019). In turn, these are used in enhanced protein glycosylation. Indeed, both the HBP and protein glycosylation are enhanced in lung and colon cancers (Mi et al., 2011). In our Kras model, disease progression appears to be accompanied by an increased demand for these UMP glycosylated substrates, as well as of 3-methylxanthine, AMP and UMP.

There was no evidence, from the levels of lactate, for increased glycolytic activity in the early stages of the disease (from N to LG), but lactate levels clearly increased throughout disease progression, as has been observed previously observed in this disease model (Serrao et al., 2016; Schmahl et al., 2018). Such an increase was particularly evident in the transition from HG to PDA, which was also consistent with a marked decrease in glucose concentration. This decrease followed a marked glucose increase in the transition from N to HG, which was also observed in a previous report of PanIN-affected 15-month old Kras mice (Schmahl et al., 2018), in which the authors suggested there was a reduction of glucose consumption in PanIN cells, compared to the normal acinar cells of healthy pancreata. The HG to PDA transition was also characterized by a set of additional statistically significant changes, including increased alanine, proline, 3-HBA and 3-HIBA concentrations and decreased ascorbate and PE concentrations. This metabolic signature may be descriptive of the HG to PDA transition and has the potential to aid diagnosis of the later stages of disease progression.

## Conclusion

We have established that Pt tissue can be differentiated with high sensitivity and specificity from N and LG tissues on the basis of their corresponding metabolite signatures. Interpretation of the accompanying metabolic changes requires consideration of the higher cellularity of Pt tissue. A 10-metabolite tissue signature (increased ADP, ATP, GSH, phenylacetate; and decreased alanine, AMP, formate, NAM, TMAO and UMP) appeared to be specific for acute Pt, differentiating this benign condition from N and LG pancreata. LG tissue (PanIN 1/1A lesions) could be distinguished from normal tissue with high statistical robustness. Changes in the relative concentrations of alanine, aspartate, creatine, glutamate, lysine, phenylalanine tyrosine and valine, *m-*inositol, PC, UDP-Glc/GlcA and UDP-GlcNAc were identified as potential markers of the precancerous stages of PCa. These reflect early changes in amino acid metabolism, membrane biosynthesis and the hexosamine biosynthesis pathway, which impacts on protein glycosylation. With progression beyond LG, changes in the concentrations of alanine, aspartate, glutamate, lysine, proline, tyrosine, valine, lactate, 3-HBA, glucose, phospholipid precursors (in particular PC) and glycosylated uridine nucleotides formed a metabolic signature that characterized PCa progression. In particular, the HG to PDA transition was accompanied by increases in alanine, 3-HBA, lactate and proline concentrations and decreases in the concentrations of ascorbate, glucose and PE. Possible interesting follow-ups of this work may include validation by absolute quantitation of the signatures’ metabolites (preferably using targeted methods to circumvent possible effects of interaction of internal standards with mixture components), and correlation studies between metabolic profiles and histological characteristics of Pt, normal and precancerous and cancerous tissues, as such information would unveil more objective hypotheses to explain the metabolic changes reported here.

## Data Availability

The datasets presented in this study can be found in online repositories. The names of the repository/repositories and accession number(s) can be found below: https://www.metabolomicsworkbench.org, Study ID ST002202 and doi http://dx.doi.org/10.21228/M83T37.
