# Supplementary material for: Metabolic profiling of induced acute pancreatitis and pancreatic cancer progression in a mutant Kras mouse model
Source: Front Mol Biosci. 2022 Aug 25;9:937865. doi: 10.3389/fmolb.2022.937865 (PMC9452780; doi:10.3389/fmolb.2022.937865)
Supplement: Supplementary file 1 [file DataSheet1.pdf]

Supplementary Material

1 Supplementary Tables

Supplementary Table 1. List of compounds and corresponding spin systems identified in the ^{1}H NMR spectra of aqueous extracts of pancreatic tissue of transgenic Kras mice. HMDB ID: human metabolome database metabolite code number. Compound abbreviations: GSH, glutathione (red.); AMP, adenosine monophosphate; ADP, adenosine diphosphate; ATP, adenosine triphosphate; NAD^{+}, nicotinamide adenine dinucleotide (reduced); UDP, uridine diphosphate; UMP, uridine monophosphate; UTP, uridine triphosphate; NAM, niacinamide; UDP-GlcNAc, uridine diphosphate *N*-acetyl-glucosamine; UDP-Glc/GlcA, uridine diphosphate glucose/ glucuronate; 3-HBA, 3-hydroxybutyrate; 3-HIBA, 3-hydroxyisobutyrate; 3-HIVA, 3-hydroxyisovalerate; TMAO, trimethylamine-*N*-oxide. s: singlet, d: doublet, m: multiplet, dd: doublet of doublets, t: triplet, q: quartet.

**Family of compounds**	**Metabolite**	**δ ^{1}H, ppm (multiplicity, assignment)**	**HMDB ID**
Amino acids and derivatives	Alanine	1.48 (d, βCH_3); 3.78 (q, αCH)	HMDB0000161
	Aspartate	2.67 (dd, βCH); 2.80 (dd, β'CH); 3.90 (dd, αCH)	HMDB0000191
	Glutamate	2.04 (m, βCH); 2.11 (m, β'CH); 2.35 (m, γCH_2); 3.74 (dd, αCH)	HMDB0000148
	Glutamine	2.13 (m, βCH_2); 2.45 (m, γCH_2); 3.77 (t, αCH)	HMDB0000641
	Glycine	3.56 (s, αCH_2)	HMDB0000123
	GSH	2.16 (m, βCH_2 Glu); 2.55 (m, γCH_2 Glu); 2.96 (m, αCH_2 Cys); 3.78 (αCH Glu); 4.16 (γCH_2 Cys); 4.57 (m, βCH_2 Cys); 8.37 (NH Gly); 8.56 (NH Cys)	HMDB0000125
	Creatine	3.04 (s, N-CH_3); 3.93 (s, N-CH_2)	HMDB0000064
	Histidine	3.10 (dd, βCH_2); 3.19 (dd, β'CH_2); 3.91 (dd, αCH); 7.08 (s, C4H ring); 7.90 (s, C2H ring)	HMDB0000177
	Isoleucine	0.94 (t, δCH_3); 1.01 (d, β'CH_3); 1.27 (m, γCH_2); 1.47 (m, γ'CH_2); 1.99 (m, βCH); 3.67 (d, αCH)	HMDB0000172
	Leucine	0.96 (t, δCH_3/ δ'CH_3); 1.71 (m, βCH_2/ γCH); 3.73 (t, αCH)	HMDB0000687
	Lysine	1.45 (m, γCH_2); 1.70 (m, δCH_2); 1.89 (m, βCH_2); 3.01 (t, εCH_2); 3.74 (t, αCH)	HMDB0000182
	Phenylalanine	3.19 (m, βCH_2); 3.99 (dd, αCH); 7.32 (d, C2H/ C6H ring); 7.38 (m, C4H ring); 7.42 (t, C3H/ C5H ring)	HMDB0000159
	Proline	1.99 (m, γCH_2); 2.07 (m, βCH_2); 2.35 (m, β'CH_2); 3.34 (m, δCH_2); 3.41 (m, δ'CH_2); 4.12 (dd, αCH)	HMDB0000162
	Taurine	3.26 (t, S-CH_2); 3.42 (t, N-CH_2)	HMDB0000251
	Threonine	1.33 (d, γCH_3); 3.58 (d, αCH); 4.24 (m, βCH)	HMDB0000167
	Tyrosine	3.06 (m, β'CH_2); 3.20 (m, βCH_2); 3.95 (m, αCH); 6.90 (d, C3H/ C5H ring); 7.20 (d, C2H/ C6H ring)	HMDB0000158
	Valine	0.99 (d, γCH_3); 1.05 (d, γ'CH_3); 2.27 (m, βCH); 3.61 (d, αCH)	HMDB0000883
Phospholipid precursors	Choline	3.21 (s, $N(CH_3)_3$); 3.53 ($C\underline{H}_2$)NH)); 4.07 (m, $C\underline{H}_2$(OH))	HMDB0000097
	Glycerophosphocholine	3.23 (s, $N(CH_3)_3$); 3.62 (m, γCH_2); 3.90 (m, βCH/ β'CH2 (N)); 3.94 (m, αCH_2); 4.33 (m, PO_3-αCH_2)	HMDB0000086

	Phosphocholine	3.22 (s, $N(CH_3)_3$); 3.59 (m, N-CH_2); 4.17 (m, PO_3-CH_2)	HMDB0001565
	Phosphoethanolamine	3.21 (t, N-CH_2); 3.98 (m, PO_3-CH_2)	HMDB0000224
Sugars	α-Glucose	3.41 (t, C5H); 3.53 (dd, C2H); 3.71 (t, C3H); 3.76 (dd, C6H); 3.83 (m, C4H); 3.83 (m, C6H'); 5.23 (d, C1H)	HMDB0003345
	β-Glucose	3.23 (dd, C2H); 3.40 (t, C5H); 3.47 (dd, C4H); 3.49 (t, C3H); 3.71 (dd, C6H'); 3.89 (m, C6H); 4.64 (d, C1H)	HMDB0003345
	Sucrose	3.48 (t, C4H); 3.56 (dd, C2H); 3.68 (s, C1'H_2); 3.76 (t, C3H); 3.82 (m, C6'H, C6H); 3.87 (m, C5H); 3.89 (m, C5'H); 4.05 (t, C4'H); 4.22 (d, C3'H); 5.42 (d, C1H)	HMDB0000258
Nucleotides and derivatives	AMP	4.01 (dd, C5'H_2 ribose), 4.36 (dd, C4'H ribose), 4.50 (dd, C2'H ribose), 6.14 (d, C1'H ribose), 8.27 (s, C8H ring), 8.60 (s, C2H ring)	HMDB0000045
	ADP	4.22 (m, C5'H ribose); 4.38 (m, C4'H ribose); 4.61 (m, C2'H ribose); 6.15 (d, C1'H ribose); 8.28 (s, C8H ring); 8.54 (s, C2H ring)	HMDB0001341
	ATP	4.22(m, C5'H ribose); 4.30 (m, C5''H ribose); 4.40 (m, C4'H ribose); 4.61 (m, C2'H ribose); 6.14 (d, C1'H ribose); 8.23 (s, C2H ring); 8.53 (s NH ring)	HMDB0000538
	NAD^+	4.23 (m, A5'); 4.36 (m, A4'); 4.39 (m, A4'/N5'); 4.42 (dd, N3'); 4.50 (m, A3'); 4.54 (m, N2'); 6.04 (d, N1'); 6.10 (d, A1'); 8.18 (s, A2); 8.19 (N5); 8.43 (s, A8); 8.83 (d, N4); 9.14 (d, N6); 9.34 (s, N2)	HMDB0000902
	NAM	7.60 (dd, C5H ring); 8.25 (dd, C4H ring) 8.72 (dd, C6H ring); 8.94 (s, C2H ring)	HMDB0001406
	UDP	4.23 (m, C5'H ribose); 4.27 (m, C4'H ribose); 4.40 (t, C2'H ribose); 4.44 (t, C3'H ribose); 5.97 (s, C1'H ribose); 5.98 (d, C6H ring); 7.97 (d, C5H ring)	HMDB0000295
	UMP	4.01 (m, C5'H_2 ribose); 4.29 (m, C4'H ribose); 4.37 (t, C3'H ribose); 4.43 (t, C2'H ribose); 5.99(m, C6H ring); 8.10 (d, C5H ring)	HMDB0000288
	Uridine	3.88 (dd, C5'H ribose); 4.23 (t, C3'H ribose); 4.38 (t, C2'H ribose); 5.90 (d, C5H ring); 5.94 (d, C1'H ring); 7.86 (d, C6H ring)	HMDB0000296
	UTP	4.26 (m, C5'H ribose); 4.30 (m, C4'H ribose); 4.42 (t, C2'H ribose); 4.45 (t, C3'H ribose); 5.97 (s, C1'H ribose); 5.99 (d, C6H ring); 7.98 (d, C5H ring)	HMDB0000285
	UDP-GlcNAc	5.52 (dd, C1H acetylglucosamine)	HMDB0000290
	UDP-Glc/GlcA	5.61(dd, C1H Glc); 7.95(d, C2H Uridine)	HMDB0000286 HMDB0000935
Organic acids	2-Phosphoglycerate	4.43 (m)	HMDB0000362
	3-HBA	1.20 (d, CH_3); 2.31 (m, CH_2); 2.41 (m, CH_2); 4.16 (m, CH)	HMDB0000357
	3-HIBA	1.08 (d, CH_3); 2.64 (m, CH); 3.68 (m, CH_2)	HMDB0000023
	3-HIVA	1.25 (s, CH_2); 2.35 (s, CH_3)	HMDB0000754
	Acetate	1.92 (s, βCH_3)	HMDB0000042
	Ascorbate	3.76(m, CH_2(OH)); 4.02 (m, CH(OH)); 4.51 (d, C1H)	HMDB0000044
	Formate	8.46 (s, CH)	HMDB0000142
	Fumarate	6.52 (s, CH)	HMDB0000134
	Lactate	1.33 (d, CH_3); 4.10 (q, CH)	HMDB0000190
	Phenylacetate	3.53 (s, CH_3); 7.26 (m, C2H, C6H, C4H ring); 7.37 (m, C3H, C5H ring)	HMDB0000209
	Succinate	2.41 (s, CH_2)	HMDB0000254
Other compounds	3-Methylxanthine	3.52 (s, CH_3), 8.03 (s, CH)	HMDB0001886

Creatinine	3.05 (s, NCH_3); 3.94 (s, NCH_2)	HMDB0000562
m-Inositol	3.28 (t, C5H); 3.54 (dd, C1H, C3H); 3.62 (t, C4H, C6H); 4.06 (t, C2H)	HMDB0000211
Methanol	3.36 (s, CH_3)	HMDB0001875
TMAO	3.27 (s, CH_3)	HMDB0000925

Supplementary Table 2. Quality parameters obtained for pairwise PLS-DA models through MCCV calculations: median predictive power or Q^2_{median}, % classification rate (CR), % sensitivity and % specificity, in comparison with single model predictive power, $Q^2_{(single\ model)}$. Values in brackets correspond to models with no or poor classification capability.

PLS-DA comparisons	$Q^2_{(single\ model)}$	Q^2_{median}	CR %	Sensivity %	Specificity %
Pt *vs* N	0.97	0.67	93	92	93
Pt *vs* LG	0.87	0.79	99	100	99
LG *vs* N	0.74	0.29	86	78	93
HG *vs* LG	0.64	< 0	(71)	(65)	(75)
PDA *vs* HG	< 0.5	< 0	(66)	(55)	(75)
Sarc *vs* PDA	< 0.5	< 0	(60)	(73)	(39)

2 Supplementary Figures

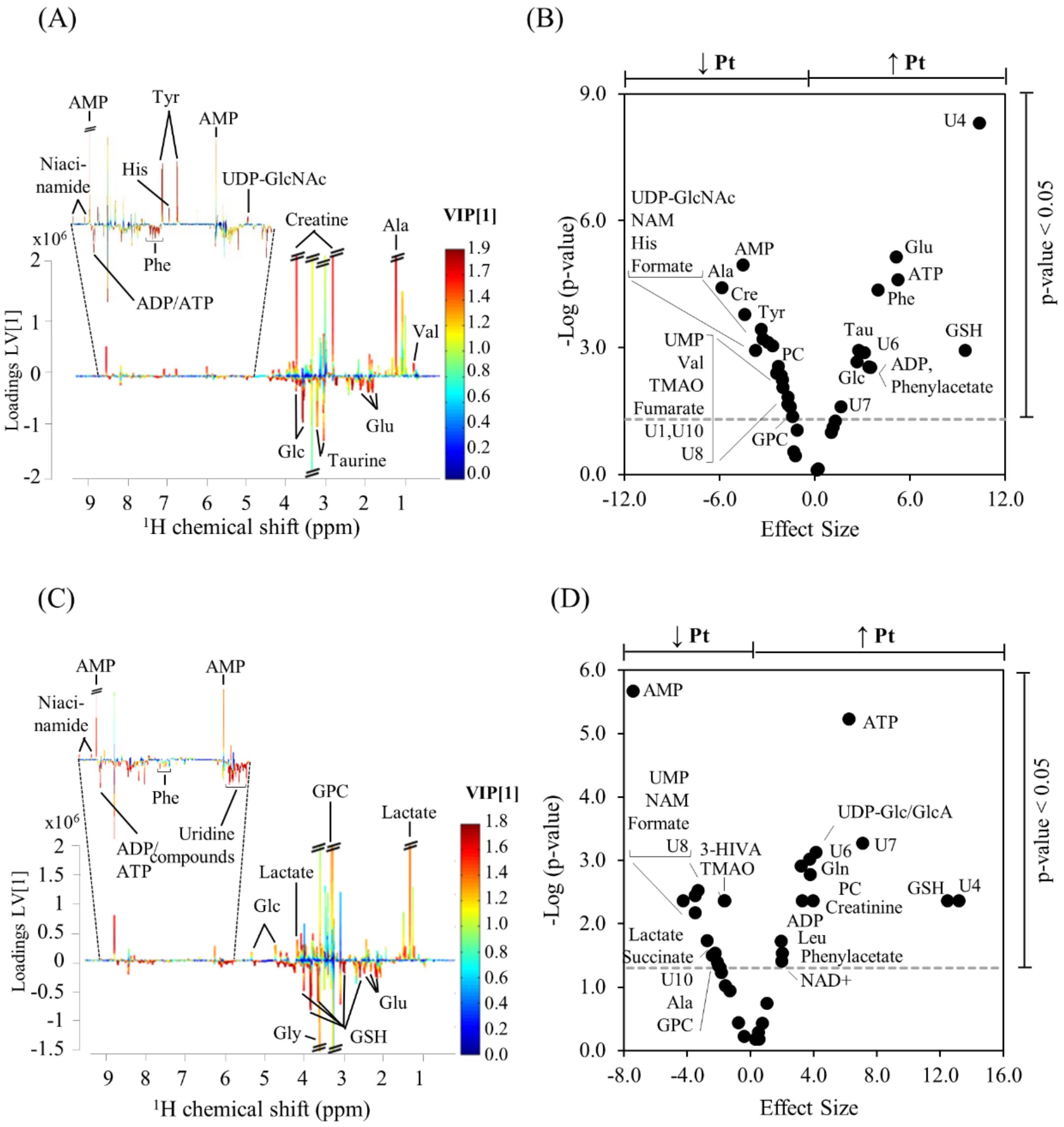

Supplementary Figure 1. PLS-DA LV1 loading plots (A, C) and Volcano plots (effect size as a function of –log[p-value]) (B, D), obtained for Pt compared to Normal tissue (A, B) and compared to LG PanIN lesions (C, D). All compound abbreviations are defined as in Figure 1 and Table 2. Three-letter code used for amino acids.
